# Prevalence and Genotyping of Torque Teno Virus in HBV/HIV and Chronic HBV Patients in Iran

**DOI:** 10.29252/ibj.22.5.338

**Published:** 2018-09

**Authors:** Zahra Najafimemar, Alijan Tabarraei, Gholamreza Talei, Abdolvahab Moradi

**Affiliations:** 1 Infectious Diseases Research Centre, Golestan University of Medical Sciences, Gorgan, Iran; 2 Department of Microbiology, Faculty of Medicine, Lorestan University of Medical Sciences, Lorestan, Iran; 3 Department of Microbiology, Faculty of Medicine, Golestan University of Medical Sciences, Golestan, Iran

**Keywords:** HIV, Iran, Polymerase chain reaction, Torque teno virus

## Abstract

**Background:**

Torque teno virus (TTV) was the first human Anelloviridae detected in a Japanese patient with an unknown type of hepatitis in 1997. TTV is by far the first known single-stranded circular DNA virus infecting human. In spite of its widespread nature in human population, its pathogenesis is still unclear. In addition, information regarding TTV infection in Iranian population is limited. Therefore, we attempted to determine the prevalence and genotype of

**TTV in three groups:**

HIV/HBV patients, chronic hepatitis B patients, and healthy individuals.

**Methods:**

The presence of TTV DNA in sera was investigated using PCR. The primer sets encompassing two 5’-UTR and N22 regions were used, and the positive products were collected for sequencing. Phylogenetic tree was generated based on N22 region and using the MEGA 7 software.

**Results:**

TTV DNA was detected in 452 patients with HIV/HBV and chronic hepatitis B, as well as in healthy control groups. The results from PCR indicated positive rates for these three groups 48%, 54%, and 49.3% using 5’-UTR primer and 15.1%, 12%, and 8% using N22 primer, respectively.

**Conclusion:**

Five genogroups were observed, which the second group was found to be the most frequent. The results of 5’-UTR primer showed more prevalence of TTV DNA comparing to N22 primer in patients and healthy control.

## INTRODUCTION

Human torque teno virus (TTV) is a recent identified infectious agent that has been assigned to the Anelloviridae family[1]. The first TTV was isolated from the serum of a patient with post-transfusion hepatitis of unknown etiology in 1997[2]. It has a negative-sense, single-stranded circular DNA genome of approximately 3.8 kb and an unenveloped, small, spherical particle with a diameter of 50 ± 30 nm[2]. Sequence diversity of TTV is extremely wide, which is classified into at least 23 genotypes with sequence divergence of more than 30% for into five phylogenetic groups[2]. These phylogenic groups have a very high prevalence in the general population[1]. TTV can be transmitted through blood transfusions, intravenous drugs, and fecal oral routes. TTVs co-infections with HIV or HBV are common since they share similar transmission routes as HIV and HBV[3]. Since the discovery of human Anelloviruses, TTV has been suggested to be associated with various pathological conditions, such as hepatitis, cancer, respiratory diseases, as well as hematological and autoimmune disorders, with few arguments for their direct involvement[1]. Three strains of TTVs recently shown to more infect humans[4] are reclassified in Anellovirus as well[5,6]. Detection of TTV is mainly performed by PCR. The PCR targeting the N22 region within the largest open reading frame 1 (ORF1) can mainly detect 1 to 5 TTVs in 10%–30% of the population, while the PCR directed for the UTR region can find most known TTVs in over 90% of the population[6,7].

TTV has been reported to be associated with hepatitis of unknown etiology[6]. Nucleotide sequence variations between TTV genotypes can be more than 50%, which leads to 39 genotypes[5]. Before the discovery of TTV, nohuman virus with such a small genome accompanied with a high mutation rate has been reported. TTV genome variation is higher at ORF levels than in the UTRs regions[5]. In a study, Takayama *et al*.[8] analyzed 50 sera samples from hemophilic patients for TTV DNA with 44.4% detection rate. The N22 gene of the virus was present in 1.9% to 42.4% of blood donors in different countries, and for UTR gene, the rates were 69% to 94%[9-14]. The goal of this study was to determine the prevalence and genotyping of TTV in Iran among three groups: HIV/HBV patients, chronic hepatitis B patients, and healthy individuals.

## MATRIALS AND METHODS

### Patients

Subjects for this study consisted of 152 HIV/HBV co-infected patients, 150 chronic HBV patients, as well as 150 uninfected individuals who were collected from Iran between April 2012 and February 2014. Samples related to HIV/HBV co-infected patients were gathered by collaboration with Lorestan University of Medical Sciences (Lorestan, Iran). However, samples regarding chronic HBV patients were obtained from patients referred by a specialist in HBV infection diagnosis to Virology Diagnostic Laboratory of Golestan University of Medical Sciences (Golestan, Iran). All patients were tested for serological marker of HBV (HBsAg) using a commercial ELISA kit from DIALAB (Austria), and Western blotting was employed as a serological test for HIV detection. Sera were separated from a total of 452 blood samples and kept in -70 °C for further analysis. All the samples were tested by PCR on the virus N22 and 5’-UTR regions. The study was approved by the Ethics Committee of Golestan University of Medical Sciences under ethic no. Ir. goums.REC.1394.102.

### DNA preparation and primer selection

DNA was extracted from all samples using Qiagen DNA Extraction Kit (Qiagen, Iran). The forward and reverse primer sequences used for 5’-UTR were 5’-GCTACGTCACTAACCACGTG-3’ and 5’-CTBC GGTGTGTAAACTCACC-3’ and for N22 included 5´-ACAGACAGAGGMGAAGGMAAY ATG-3´ and 5´-CTGGCATTTTACCATTTCCAAAGT T-3´, respectively. The amplicons predicted for 5’-UTR and N22 regions found to be 204 bp and 271 bp, respectively[6,15].

### Detection of TTV with N22 region primers

**Fig. 1:**
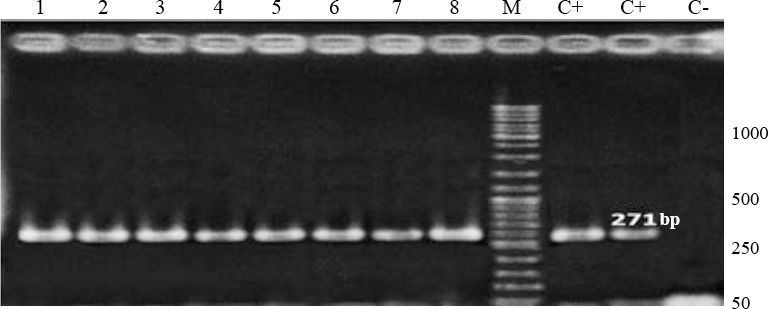
Identification of Torque teno virus (TTV) by PCR amplification of N22 gene. Lanes 1 to 8, positive samples of TTV; lanes C+, positive control samples; lane C-, negative control sample. M, 50 bp DNA ladder

The TTV PCR reaction for N22 region contained 100 ng of template DNA, 1 μL (15 pM stock) of each amplification primer, 1 μL of dNTP (10 mM stock), 0.5 μL of Taq DNA polymerase (GeNet Bio, Korea), 2 mM of MgCl_2_, and 2.5 μL of 10× PCR buffer. The following thermal cycles were set for genome amplification: initial denaturation at 94 °C for 7 min, followed by 35 cycles at 94 °C for 30 s, 60 °C for 40 s, and 72°C for 40 s, with a final elongation at 72 °C for 7 min. The PCR products were separated on 1.5% agarose gel containing safe stain, Eva green (Cinaclone, Iran; Fig. 1).

### TTV detection based on UTR region amplification

**Fig. 2:**
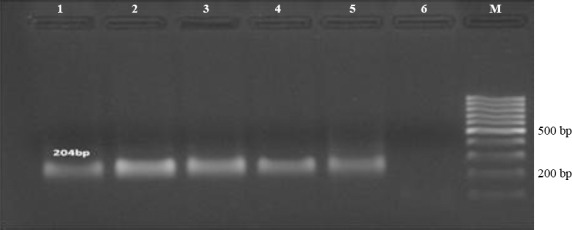
Identification of Torque teno virus (TTV) by PCR amplification of the 5’UTR region. Lanes 1 to 4, positive samples of TTV; lanes 5 and 6, positive and negative controls, respectively. M, 100 bp DNA ladder

The PCR reaction for 5’-UTR region included 100 ng of template DNA, 10 pmol of each forward and reverse primer, 0.6 mM of dNTPs, 1.25 U of Taq DNA polymerase (GeNet Bio), 2 mM of MgCl_2_, and 1× PCR buffer. Thermal amplifications were as follows: initial denaturation at 94 °C for 5 min and 40 cycles at 94 °C for 20 s, 57 °C for 25 s, and 72 °C for 30 s, and a final extension at 72 °C for 5 min (Fig. 2).

### Sequence data mining

In total, 44 DNA sequences of TTV isolates were obtained from the GenBank database and used as a reference sequences for further analysis.

### Phylogenetic analysis

Phylogenetic tree was constructed based on TTV N22 amplicon sequenced data and compared to the TTV reference sequence with accession number AB008394. Before comparison, the sequenced data were trimmed and aligned by BioEdit (5.0.9) and ClustalW (1.81) software, respectively. Bootstrap resampling was carried out on 1000 replicates to ensure the reliability of the tree.

## RESULTS

By using 5’-UTR primers, TTV-DNA was detected in 48% (73/152) patients with HIV/HBV co-infection, in 56% (84/152) with chronic hepatitis B infection, and in 49.3% (74/152) healthy blood donors. Meanwhile, 15.1% (23/152) of patients with HIV/HBV co-infection, 12% (18/152) with chronic hepatitis B, and 8% (12/152) of healthy blood donors were observed to be positive for N22 primers. The positive samples for N22 were also observed to be positive by 5’-UTR primer. A total of 15 samples were randomly selected from all the positive products of 271 nt from the N22 region and were successfully sequenced.

**Fig. 3:**
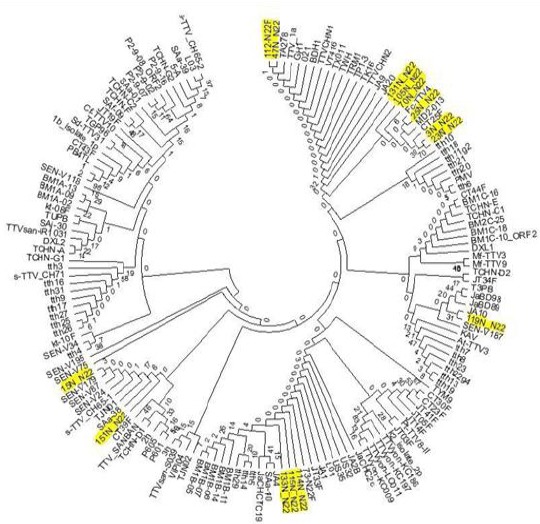
Phylogenetic tree of human Anelloviruses. The tree was built using the MEGA 7 software with the neighbor-joining method[16] of the nucleotide sequence of ORF1 in 15 TTV isolates. The phylogenic tree includes 140 isolates obtained from the International Committee on Taxonomy of Viruses (ICTV). Available complete ORF1 sequences of TTV were downloaded from the GenBank with accession number AB008394. The isolated viruses in the present study are highlighted in yellow.

Based on phylogenetic analysis, 5 genogroups (1, 2, 3, 4, and 5) were identified. The analysis of 15 N22 sequenced isolates demonstrated that genogroup 2 was significantly more prevalent among all the patients (Fig. 3). Also, the five genotypes were found among HIV/HBV, chronic HBV patients, and uninfected individuals in Iran. Demographic and paraclinical data are shown in Tables 1 and 2.

**Table 1:** General features of the TTV groups under study

Groups	Gender	Age (y)			*p* value
	
	Female/male ration	1-20	21-40	41-60	
HIV/HBV (n = 152)	50/102	4	96	52	
TTV + for gene 5’-UTR	73 (48%)				0.1
TTV + for gene N22	23 (15.1%)				0.0003
CHBV (n = 150)	42/108	1	120	29	
TTV + for gene 5’-UTR	84 (56%)				0.1
TTV + for gene N22	18 (12%)				0.2
Healthy control (n =150)	80/70	10	81	59	
TTV+ for gene 5’-UTR	74 (49.3%)				0.1
TTV+ for gene N22	12 (8%)				0.2

**Table 2:** TTV genogroups observed in different studied groups

Genogroup/isolated virus	HIV/HBV	CHBV	Healthy control
**1**			
EG1	*		
Isfahan MEF6	*		
T17-12VHC	*		
Isfahan MEF6		*	
**2**		*	
YOGHttv55			
Sle2001		*	
TCHN-G1	*		
NZA330	*		
G75		*	
**3**			*
US47			
YOGHttv22	*		
**4**	*		
NZA330			
HRpd268	*		
HRpd254		*	
**5**	*		
Isfahan MEF8			

* TTV seen in this genogroup

## DISCUSSION

TTV was first reported in Japan in 1997 by Nishizawa in patients with fulminant hepatitis and chronic liver disease of unknown etiology. This virus was later identified in a large number of patients, who mostly suffered from acute and chronic hepatitis, in several countries[17,18]. The association between TTV infection and hepatitis is argumentative as there is no evidence of causative effect of TTV to date[19-21].

Concomitant infections with TTV and HBV are common; nonetheless, the effect of TTV infection on patients with chronic HBV infection is unknown[22]. TTV has been found to be highly common in men worldwide[23]. Currently, only PCR assay is available for TTV virus[24], but the accurate detection of all TTV genotypes depends on the selection of the amplification region. Current primers are designed for either N22 or 5’-UTR regions. N22 primers only recognize and amplifiy some of the genotypes, but 5’-UTR primers are able to identify and amplify all types of virus genotype[25]. Here, we showed that TTV infection cannot be frequently detected by 5’-UTR primers in chronic HBV patients. Based on our results, it can be concluded that both N22 and 5’-UTR primers can detect the TTV infection; however, the prevalence may be in a wide range of 5 to 92% due to the sensitivity of the primers. Several studies support this wide range of the reported prevalence of TTV in a single population based on the applied primers.

Conservation of 5’-UTR locus within different genotypes may explain the high incidence of TTV detection based on 5’-UTR primers. It was generally observed that the prevalence of TTV infection based on N22 region amplification was below 15% in healthy subjects. The N22-based PCR method generally had a lower detection rate than UTR PCR, due to its limited genotype specificity. This finding has also previously been reported by Ergünay group[26]. TTV prevalence among Brazilian blood donors, by using different primers, was reported to be 11.9% to 50.5%. Accordingly, TTV prevalence in Saudi Arabia has been reported to be up to 100%, when 5’-UTR primers were used[27]. Because of a very high genetic variety, several studies have been conducted to investigate the prevalence of TTV. These studies are affected by the viral, amplified region and sensitivity of its primers[28]. Phylogenetic analysis of TTV was firstly performed on N22 region, as it was suggested that this region would exploit enough diversity for phylogenetic analysis. However, TTV genome amplification has revealed higher genetic variety than other DNA viruses.

Therefore, TTV genotyping within the N22 region resulted in inaccurate observations. At the present study, some clinical parameters such as sex and age was evaluated. The results indicated no correlation between TTV infection and sex and age among the three groups under study. However, a significant correlation was observed between gene N22 of TTV and sex (women with HIV/HBV infection; *p* < 0.004).

The results of the present study in comparison with other reports demonstrate that different primers chosen in studies could affect the findings. To date, there is little or no data covering the incidence and genotype distribution of human TTV in Iranian population afflicted by HIV/HBV co-infections, CHBV infection, and or healthy individuals. To achieve this goal, further investigations with larger population samples are needed. Accordingly, the patients have to be selected from different parts of the country.

Likewise the previous studies, the present work shows that the 5’-UTR primer-based PCR may report more prevalence of TTV DNA comparing to N22 primers either in patients or healthy control (Tables 3 and 4).

**Table 3:** Comparison of N22 gene frequency in different studies

Country	Method	Percentage of TTV prevalence	Year
Iran[29]	semi-nested PCR	26.9% (liver tissue); 23.5% (plasma samples of transplanted patients with cryptogenic cirrhosis); 25.9% (liver tissue), 11.1% (plasma samples of patients with determined cirrhosis)	2015
Iran[30]	Nested and semi-nested PCR/RFLP	21.33% (healthy blood donors); 43.33% (HIV patients)	2014
Iran[31]	PCR	8.9% (HBV); 209% (healthy control)	2011
Brazil[32]	Nested PCR	12.5% (HIV), 6% (healthy control)	2009
India[33]	RFLP	26.7% (liver diseases); (58.5%) chronic renal failure	2008
Turkey[26]	PCR	11.8% (cryptogenic hepatitis); 11.8% (HBs carriers); 16.7% (chronic HBV hepatitis); 2.5% (controls)	2008
Iran[34]	Semi-nested PCR	9.3% (patients on maintenance hemodialysis)	2007
Egypt[35]	Nested PCR	23.8% (chronic hepatitis); 31.8% (hepatocellular carcinoma)	2006
Turkey[36]	PCR	19.1% (chronic HBV hepatitis); 30.4% (healthy blood donors)	2006

**Table 4:** Comparison of 5’UTR gene frequency in different studies

Country	Method	Percentage of TTV prevalence	Year
Iran[30]	PCR	64% (HIV); 34% (healthy control)	2014
Iran[37]	Semi-nested PCR	18% (HBV); 50.8% (healthy control)	2013
Pakistan[38]	Nested PCR	92.5% (HBV); 89.7% (healthy control)	2012
India[39]	Nested PCR	73.6% (acute hepatitis); 59.2% (fulminant hepatitis)	2010
Russia[40]	PCR	94% (olympic athletes)	2009
Turkey[26]	PCR	58.8% (cryptogenic hepatitis); 47.1% (HBs carriers); 61.1% (chronic HBV hepatitis); 45% (controls)	2008
Turkey[36]	PCR	42.9% (chronic HBV hepatitis); 50% (healthy blood donors)	2006
Brazil[41]	PCR	54% (HBV); 100% (HIV); 46% (healthy control)	2004

Although our results suggested that TTV incidence was higher among HIV/HBV and chronic HBV patients comparing healthy population, TTV frequency was not significantly different between HIV/HBV co-infection and chronic HBV cases. Further investigations are needed to evaluate the risk of TTV infection in those patients.
